# The Relationship Between Irritable Bowel Syndrome and Metabolic Syndrome: A Systematic Review and Meta‐Analysis of 49,662 Individuals

**DOI:** 10.1002/edm2.70041

**Published:** 2025-03-24

**Authors:** Yomna E. Dean, Jose J. Loayza Pintado, Samah S. Rouzan, Lucy L. Nale, Ahmed Abbas, Abdulla Aboushaira, Farah Alkasajy, Ahmed A. Ghanem, Vinayak Mahesh Patil, Yana Gordeyeva, Karam R. Motawea, Masako Lien Petty Le, Adham Galal, Laura Cicani, Raneem Attta, Ahmed Soliman, Lamya Alzabidi, Anuj Subedi, Nikhat Anjum, Abdullah Nahedh, Tamer Mady, Yusef Hazimeh, Hossam Amin, Hani Aiash

**Affiliations:** ^1^ Alexandria University Faculty of Medicine Alexandria Egypt; ^2^ University of Texas Rio Grande Valley McAllen USA; ^3^ Windsor University School of Medicine Basseterre Saint Kitts and Nevis; ^4^ Zagazig University Zagazig Egypt; ^5^ Hashemite University Zarqa Jordan; ^6^ Mansoura University Faculty of Medicine Mansoura Egypt; ^7^ Karnataka Institute of Medical Science Hubli India; ^8^ Poznan University of Medical Sciences Ponzan Poland; ^9^ International University of Health Sciences Basseterre Saint Kitts and Nevis; ^10^ Ain Shams University Faculty of Medicine Cairo Egypt; ^11^ Faculty of Medicine and Health Sciences Sana'a University Sana'a Yemen; ^12^ Prithvi Narayan Community Hospital Gorkha Nepal; ^13^ Khaja Banda Nawaz Institute of Medical Sciences Gulbarga India; ^14^ International American University College of Medicine Castries Saint Lucia; ^15^ Lebanese University Beirut Lebanon; ^16^ Ben Sinai Medical Center New York New York USA; ^17^ SUNY Upstate Medical University Syracuse New York USA

**Keywords:** hypertension, insulin resistance, irritable bowel syndrome, metabolic syndrome, obesity

## Abstract

**Background:**

Studies have shown mixed results regarding the association between irritable bowel syndrome (IBS) and metabolic syndrome (MS); This study aimed to assess the susceptibility of IBS patients to MS and its individual components.

**Methods:**

PubMed, Scopus, Embase, and Web of Science were searched on 1/1/2023. Eligible studies were screened, and data on study characteristics, IBS diagnostic criteria, and metabolic syndrome components were extracted. Data were analysed in RevMan 5.4, with results reported as relative risk (RR) or mean difference (MD) and 95% confidence intervals. Statistical significance was set at *p* < 0.05.

**Results:**

IBS was associated with an increased risk of MS (RR = 2.05, 95% CI = 1.50–2.79, *p* < 0.00001), with a higher risk among IBS‐D patients (RR = 3.09, 95% CI = 2.41–3.97, *p* < 0.00001). IBS patients showed increased HOMA‐IR (MD = 0.21, 95% CI = 0.15–0.26, *p* < 0.00001), higher obesity risk (RR = 1.46, 95% CI = 1.10–1.93, *p* = 0.009), elevated BMI (MD = 1.51, 95% CI = 0.98–2.03, *p*‐value < 0.00001), waist circumference (MD = 5.01, 95% CI = 1.29–8.72, *p* = 0.008), and an association with systolic hypertension (MD = −0.50, 95% CI = −0.60 to −0.40, *p*‐value < 0.00001). IBS was also linked to higher LDL (MD = 5.98, 95% CI = 0.91–11.05, *p* = 0.02), total cholesterol (MD = 12.21, 95% CI = 6.23–18.18, *p* < 0.0001), and triglycerides (MD = 11.93, 95% CI = 11.55–12.31, *p* < 0.00001).

**Conclusions:**

This analysis indicates a potential association between IBS and metabolic syndrome, including its components such as obesity, hypertension, and lipid profile abnormalities. However, significant heterogeneity among studies limits the generalisability of these findings. Clinicians should remain aware of the possible link and consider individualised preventive and management strategies.

## Graphical abstract

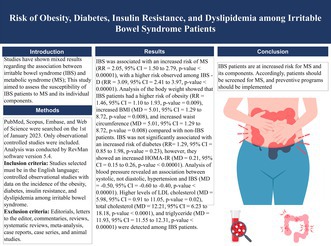

## 1 Introduction

Irritable bowel syndrome (IBS) affects approximately 9%–23% of the world population and accounts for 12% of primary care visits [1]. The precise pathogenesis of this condition is still not fully understood; Numerous mechanisms have been proposed, including dysmotility, heightened visceral sensation, disrupted brain‐gut interaction, and psychosocial distress [2, 3]. Due to its high prevalence, significant impact on the quality of life, and lack of curative therapy, irritable bowel syndrome has the capacity to exert substantial economic strain on the healthcare system [4, 5, 6].

The metabolic syndrome is a complex of interrelated risk factors for cardiovascular disease (CVD) and diabetes. These factors include dysglycemia, raised blood pressure, elevated triglyceride levels, low high‐density lipoprotein cholesterol levels, and obesity (particularly central adiposity) [7]. Remarkably, metabolic syndrome has emerged as a global epidemic, with prevalence rates reaching as high as 40% in certain regions [8] Substantial evidence indicates that dietary factors play a pivotal role in preventing and treating metabolic syndrome [9].

IBS can influence dietary patterns, food digestion, and nutrient absorption, all of which are crucial factors in preventing and treating metabolic syndrome (MS) and its individual components [10, 11]. As a result, there is speculation that IBS might represent a potential risk factor for the development of MS. Nevertheless, no study has actually explored this association between IBS status and MS, as well as its components, in adult populations. This study aimed to determine whether IBS, as well as its subtypes, is associated with metabolic syndrome and/or its individual components.

## 2 Methods

This study was conducted according to Preferred Reporting Items for Systematic reviews and Meta‐Analyses (PRISMA) guidelines [12] and its protocol was registered on Prospero (CRD42023389906).

### 2.1 Search Strategy

A literature search of PubMed, Scopus, and Web of Science was conducted on the 1st of January 2023, using key terms such as ‘metabolic syndrome’ and ‘irritable bowel syndrome’, to identify studies of interest. (View the Appendix S1 for the full strategy).

### 2.2 Inclusion and Exclusion Criteria

We screened studies by titles and abstracts according to the following criteria:

### 2.3 Inclusion Criteria

The chosen studies should be written in English and fall into the category of controlled observational research, providing information about the occurrence of the metabolic syndrome among individuals with irritable bowel syndrome (IBS). This encompasses cross‐sectional, case–control, and cohort studies.

### 2.4 Exclusion Criteria

Uncontrolled observational studies, editorials, letters to the editor, commentaries, reviews, systematic reviews, meta‐analyses, case reports, case series, and studies involving animals were excluded.

When duplicate studies were encountered, preference was given to the most recent study with the largest participant pool.

### 2.5 Study Selection

Two independent reviewers (A.A. and A.M.A.) screened the studies according to our criteria. If a consensus was not achieved, a third independent reviewer (Y.E.D.) was assigned to resolve the conflict.

### 2.6 Data Extraction and Quality Assessment

Two independent extractors (J.J.L.P. and L.L.N.) screened the studies according to our criteria. If a consensus was not achieved, a third independent reviewer (Y.E.D.) was assigned to resolve the conflict.

For the baseline and summary, the following data were extracted from the eligible studies: author year, study design, country, sample size, mean age and SD, number of males and percentage, number of smoking participants and percentage, IBS diagnostic criteria, comorbidities, and a brief conclusion. Data are shown in Table 1.

**TABLE 1:** Characteristics of the included studies.

Author (year)	Study design	Country	Sample size	Age, mean (SD)	Males, *N* (%)	Smoking, *N* (%)	IBS diagnostic criteria (e.g., Rome 4 or Rome 3, etc.)	Baseline diseases (comorbidities)	Conclusion (brief)
Lee (2016)	Cross‐sectional	Korea	343	42.4 (2.7)	235 (68.5)	81 (23.8)	Rome III	HTN, DM, obesity	Significantly higher prevalences of elevated ALT level and MS in IBS patients compared to the controls
Bayrak (2020)	Cross‐sectional	Turkey	614	37.8 (12.1)	310 (50.5)	235 (38.3)	Rome IV	HTN, DM, depression, fibromyalgia, MS, anxiety disorder	Significantly increased rate of MS among IBS patients than the control subjects
Galai (2020)	Retrospective Cohort study	Israel	126	11.5 (1.2)	59 (34.2)	N/A	Rome IV	Functional abdominal pain, functional dyspepsia	Adolescents with FAP disorders have a higher prevalence of overweight/obesity compared with controls in the general population. Children with FAP and overweight/obesity, have more hospitalizations and medical treatment with PPI compared to children with FAP disorders who are of normal weight
Kaluzna (2022)	Cross‐sectional	Poland	205	24.8 (6.2)	N/A	N/A	Rome IV	PCOS, MS	No statistical diff in IBS prevalence in PCOS pts. and controls. IBS symptoms are more frequent in females. (reproductive age) than shown in previous studies. IBS‐PCOS women had low QoL score than non‐IBS‐PCOS women. Higher MS prevalence in IBS PCOS than non IBS PCOS
Khayyatzadeh (2017)	Cross‐sectional	Iran	965	14.4 (1.4)	0	N/A	Rome III	N/A	Individuals with IBS had significantly lower serum 25‐OH D concentrations
Dogan (2022)	Cross‐sectional	Turkey	487	33.7 (8.6)	112 (23)	129 (26.5)	Rome IV	HTN, DM, cardiovascular disease, hypothyroidism, depression, upper GI diseases, fibromyalgia	No statistical relationship between IBS and abdominal obesity
GUO (2014)	Cross‐sectional	japan	1096	46.2 (11.2)	850 (78)	438 (39.9)	Rome III (Japanese version)	depression	IBS was related to a higher prevalence of MS and elevated TG. The differences in dietary patterns are not likely to explain findings. Treatment of IBS may be a potentially beneficial factor for the development and prevention of MS
Lee (2015)	Cross‐sectional	Korea	336	50.1 (11.1)	173 (51.5)	N/A	Rome III	Erosive esophagitis (infectious colitis, of gastrointestinal surgery, neuropsychiatry disease)	Visceral adiposity and waist circumference is associated with an increased risk of IBS
Javadekar (2021)	Cross‐sectional	India	1040	37.3 (7.1)	801 (77)	N/A	Rome III	MS, functional GI disorders	There was no association between MS and IBS
Buscail (2017)	Cross‐sectional	France	44,350	49.7 (14.3)	9643 (21.7)	5787 (13)	Rome III	N/A	Western dietary pattern was associated with a moderate increased risk of IBS

Abbreviations: ALT, alanine transaminase; DM, diabetes mellitus; FAP, functional abdominal pain; GI, gastrointestinal; HTN, hypertension; IBS, irritable bowel syndrome; MS, metabolic syndrome; N/A, not applicable; N, number; PCOS, polycystic ovary syndrome; PPI, proton pump inhibitors; QoL, quality of life; SD, standard deviation; TG, triglyceride.

For the outcomes, the following data were extracted: metabolic syndrome, HOMA‐IR, obesity, abdominal obesity, waist circumference, BMI, diabetes, fasting blood glucose, HbA1c, hypertension, systolic blood pressure, diastolic blood pressure, HDL‐C, LDL‐C, total cholesterol, and total triglyceride.

The risk of bias was assessed utilising the Newcastle‐Ottawa scale (NOS) items, with a total score of nine points, to evaluate the quality of observational studies. We defined the observational studies with a NOS score of ≥ 7 stars as high quality and a NOS score of < 7 stars as low quality [13]. Results are shown in Table 2.

**TABLE 2:** Quality assessment of the included studies.

Author (year)	Selection				Comparability	Exposure		Total point
	(1) Representativeness of the exposed cohort	(2) Selection of the non exposed cohort	(3) Ascertainment of exposure	(4) Non‐response rate	(1) Comparability of cohorts on the basis of the design or analysis	(1) Assessment of outcome	(2) Statistical test	
Lee (2016)	0	1	2	1	1	0	1	6
Bayrak (2020)	0	1	2	1	2	1	1	8
Buscail (2017)	1	1	2	0	2	0	1	7
Lee(2015)	0	1	2	1	2	1	1	8
GUO(2014)	1	0	1	0	0	0	1	3
Javadekar (2021)	0	1	2	1	0	0	1	5
Dogan (2022)	0	1	2	1	0	1	1	6
Kaluzna (2022)	0	1	2	1	0	1	1	6
Galai (2020)	0	0	2	1	0	1	1	5
Khayyatzadeh (2017)	0	1	0	1	1	1	1	5

### 2.7 Data Analysis

Data were analysed using RevMan software, version 5.4. Sensitivity analyses were conducted in the form of leave‐one‐out tests and subgroup analyses. If no heterogeneity was observed, results were presented in a fixed effect model and a random effect model if significant heterogeneity was observed. A relative risk (RR) with a 95% confidence interval (CI) was used to present dichotomous data, while mean difference (MD) and 95% CI were used for continuous data. Results were considered significant if the *p*‐value was less than 0.05.

### 2.8 Definition of Heterogeneity

The variation or diversity in study outcomes among the studies included in the meta‐analysismay be due to different factors, such as the characteristics of the participants, study designs, the methods of analysis, or other sources of bias [14].

## 3 Results

### 3.1 Literature Search

**FIGURE 1:**
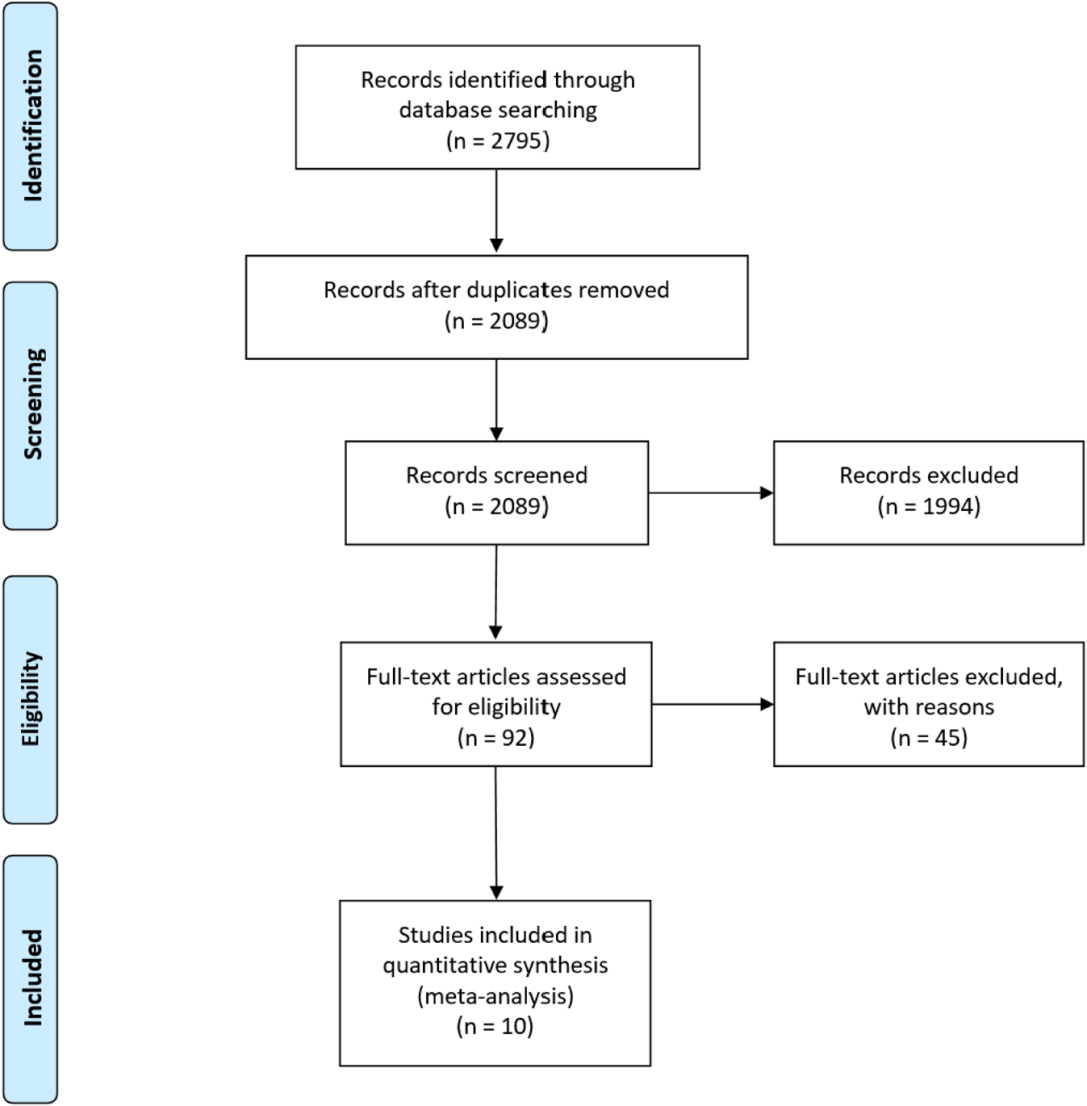
PRISMA.

2795 studies resulted after a complete literature search through database screening. After 706 duplicates were removed, 2089 studies became eligible for title and abstract screening. Of the 2089 studies, 1994 were irrelevant, and 92 studies were eligible for full‐text screening. 82 of those full‐text articles were excluded, and finally, 10 studies [15, 16, 17, 18, 19, 20, 21, 22, 23, 24] were included in the meta‐analysis after full‐text screening, as shown in the PRISMA diagram in Figure 1.

The total number of patients included in the study is 49,662 individuals, 3540 patients in the IBS group, and 46,122 patients in the control group; other baseline data are shown in Table 1. The overall quality of the included studies is shown in Table 2.

### 3.2 Outcomes

#### 3.2.1 Metabolic Syndrome Incidence

**FIGURE 2:**
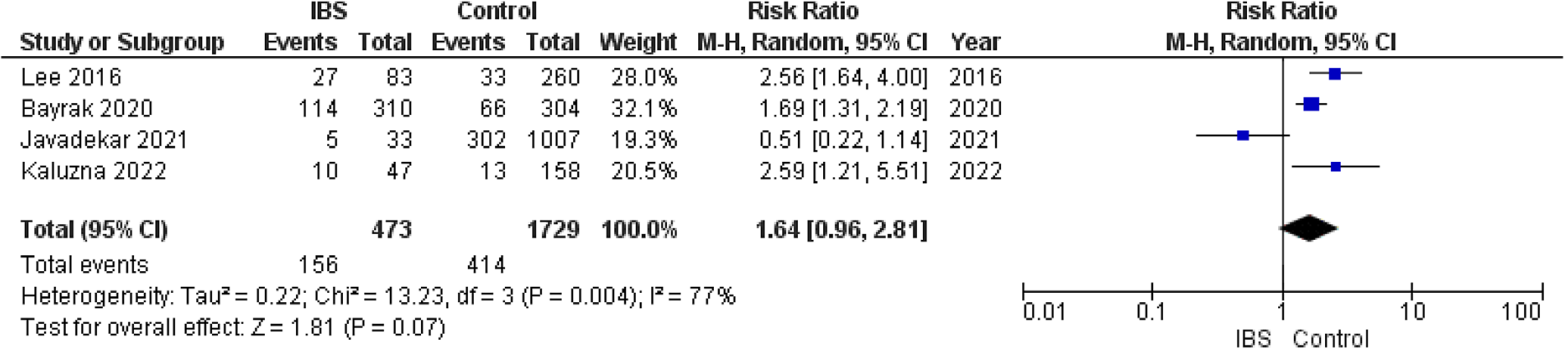
Forest plot of the comparison between the IBS group and control group in incidence of metabolic syndrome.

The pooled analysis showed no statistically significant difference between the IBS group and the control group regarding the incidence of metabolic syndrome (RR = 1.64, 95% CI = 0.96 to 2.81, *p*‐value = 0.07). We observed a significant heterogeneity between the studies (*p* = *0.004*, *I* ^*2*^ = 77%). It was solved by the leave‐one‐out test by removing Javadekar (2021) (*p* = 0.21, *I* ^*2*^ = 36%), and the analysis showed a statistically significant association between IBS and metabolic syndrome. The risk of MS among IBS patients was twice the risk in the control group (RR = 2.05, 95% CI = 1.50–2.79, *p*‐value < 0.00001). Figure 2.

### 3.3 Metabolic Syndrome Incidence by IBS Subgroups

#### 3.3.1 IBS—Constipation Subgroup

**FIGURE 3:**
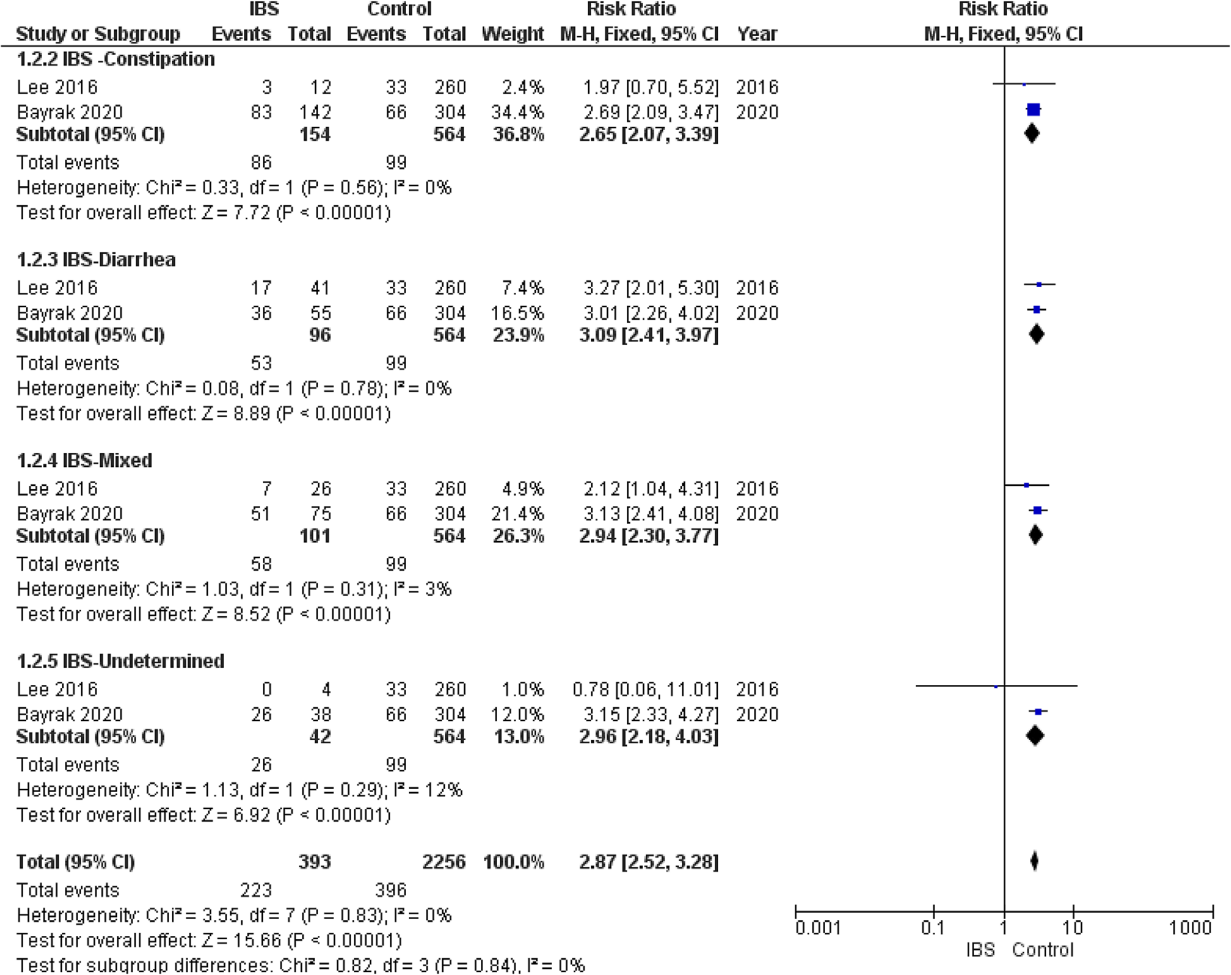
Forest plot of the comparison between the IBS group and control group in incidence of metabolic syndrome subgroups.

In the IBS–constipation subgroup, the pooled analysis showed a statistically significant association between IBS and metabolic syndrome; IBS–constipation patients had 2.65 times greater risk for MS compared to controls (RR = 2.65, 95% CI = 2.07–3.39, *p*‐value < 0.00001), with no significant heterogeneity among the studies (*p* = 0.56, *I* ^2^ = 0%). Figure 3.

#### 3.3.2 IBS—Diarrhoea Subgroup

In the IBS–diarrhoea subgroup, ; IBS–diarrhoea patients had 3.09 times greater risk for MS compared to controls (RR = 3.09, 95% CI = 2.41–3.97, *p*‐value < 0.00001), with no significant heterogeneity among the studies (*p* = 0.78, *I* ^2^ = 0%) Figure 3.

#### 3.3.3 IBS—Mixed Subgroup

In the IBS–mixed subgroup, the pooled analysis showed a statistically significant association between IBS and metabolic syndrome; IBS–mixed patients had a 2.94 greater risk for MS compared to controls (RR = 2.94, 95% CI = 2.30–3.77, *p*‐value < 0.00001), with no significant heterogeneity among the studies (*p* = 0.31, *I* ^2^ = 3%). Figure 3.

#### 3.3.4 IBS—Undetermined Subgroup

In the IBS—undetermined subgroup, the pooled analysis showed a statistically significant association between IBS and metabolic syndrome; IBS‐undetermined patients had a 2.96 greater risk for MS compared to controls (RR = 2.96, 95% CI = 2.18–4.03, *p*‐value < 0.00001), with no significant heterogeneity among the studies (*p* = 0.29, *I* ^2^ = 12%). Figure 3.

However, the test for subgroup differences showed that there were no statistically significant differences in the risk for MS between IBS subgroups (*χ* ^2^ = 0.82, df = 3, *p* = 0.84).

### 3.4 Obesity

**FIGURE 4:**
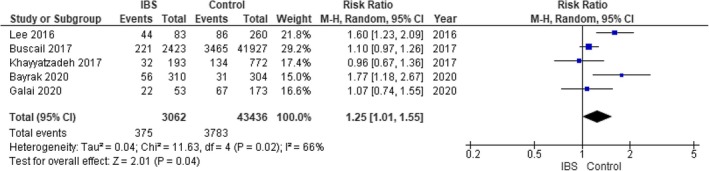
Forest plot of the comparison between the IBS group and control group in obesity outcome.

The pooled analysis showed no statistically significant difference between the IBS group and the control group regarding obesity (RR = 1.31, 95% CI = 0.98–1.75, *p*‐value = 0.07), with significant heterogeneity among the studies (*p* = 0.04, *I* ^2^ = 65%). It was solved by the leave‐one‐out test by removing Khayyatzadeh 2017(*p* = 0.13, *I* ^2^ = 50%), and the analysis showed a statistically significant association between the IBS group and increased obesity compared with the control group (RR = 1.46, 95% CI = 1.10–1.93, *p*‐value = 0.009). Figure 4.

### 3.5 Abdominal Obesity

**FIGURE 5:**
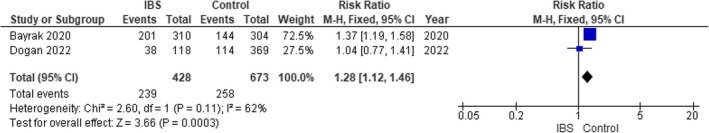
Forest plot of the comparison between the IBS group and control group in abdominal obesity.

The pooled analysis showed a statistically significant association between the IBS group and increased abdominal obesity compared with the control group (RR = 1.28, 95% CI = 1.12–1.46, *p*‐value = 0.0003), with no significant heterogeneity among the studies (*p* = 0.11, *I* ^2^ = 62%). Figure 5.

### 3.6 Waist Circumference

**FIGURE 6:**
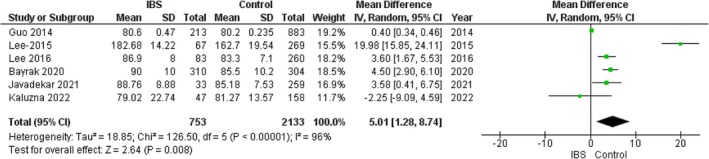
Forest plot of the comparison between the IBS group and control group in waist circumference.

The pooled analysis showed a statistically significant association between the IBS group and increased waist circumference compared with the control group (MD = 5.01, 95% CI = 1.29–8.72, *p*‐value = 0.008), with significant heterogeneity among the studies (*p* < 0.00001, *I* ^2^ = 96%). It was not solved by a leave‐one‐out test. Figure 6.

### 3.7 BMI

**FIGURE 7:**
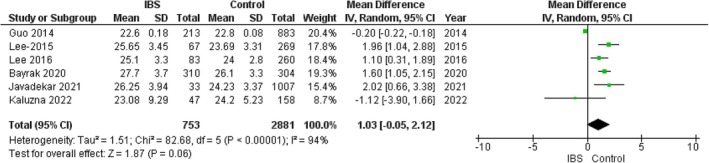
Forest plot of the comparison between the IBS group and control group in BMI.

The pooled analysis showed no statistically significant difference between the IBS group and the control group regarding BMI (MD = 1.03, 95% CI = −0.05 to 2.12, *p*‐value = 0.06), with significant heterogeneity between the studies (*p* < 0.00001, *I* ^2^ = 94%). It was solved by the leave‐one‐out test by removing Guo 2014 (*p* = 0.20, *I* ^2^ = 34%), and the analysis showed a statistically significant association between the IBS group and increased BMI compared with the control group (MD = 1.51, 95% CI = 0.98–2.03, *p*‐value < 0.00001). Figure 7.

### 3.8 BMI Subgroups

#### 3.8.1 IBS—Constipation Subgroup

**FIGURE 8:**
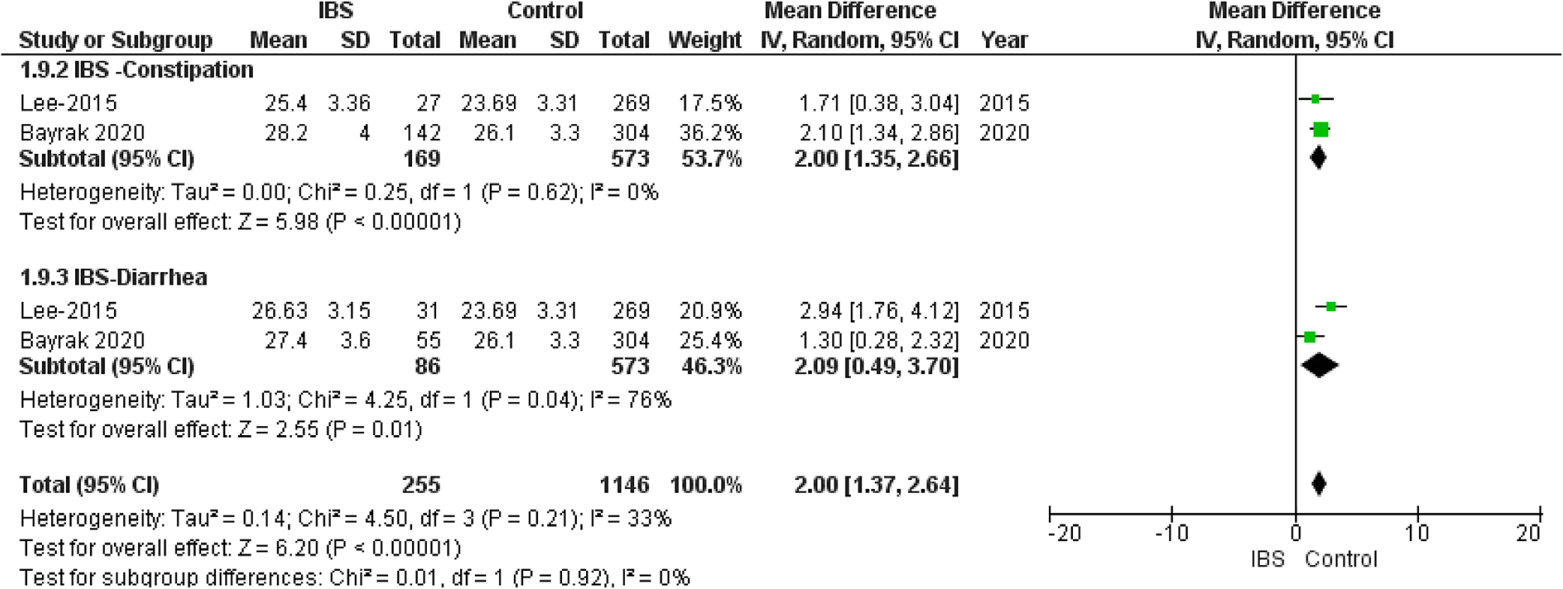
Forest plot of the comparison between the IBS group and control group in BMI subgroups.

In the IBS–constipation subgroup, the pooled analysis showed a statistically significant association between the IBS group and increased BMI compared with the control group (MD = 2.00, 95% CI = 1.35–2.66, *p*‐value < 0.00001), with no significant heterogeneity among the studies (*p* = 0.62, *I* ^2^ = 0%). Figure 8.

#### 3.8.2 IBS—Diarrhoea Subgroup

In the IBS–diarrhoea subgroup, the pooled analysis showed a statistically significant association between the IBS group and increased BMI compared with the control group (MD = 2.09, 95% CI = 0.49–3.70, *p*‐value = 0.01), with significant heterogeneity among the studies (*p* = 0.04, *I* ^2^ = 76%). Figure 8.

#### 3.8.3 Diabetes

**FIGURE 9:**
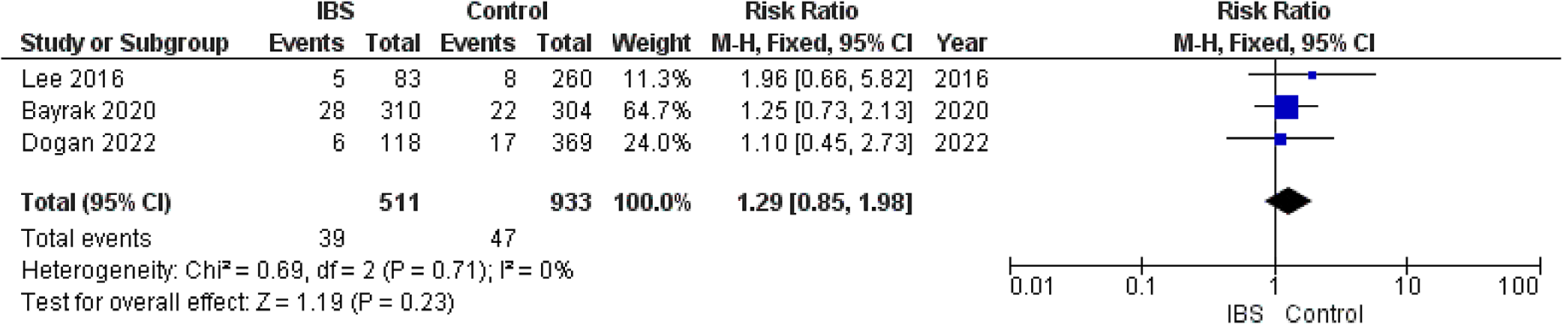
Forest plot of the comparison between the IBS group and control group in diabetes.

The pooled analysis showed no statistically significant difference between the IBS group and the control group regarding diabetes (RR = 1.29, 95% CI = 0.85–1.98, *p*‐value = 0.23), with no significant heterogeneity among the studies (*p* = 0.71, *I* ^2^ = 0%). Figure 9.

#### 3.8.4 Fasting Blood Glucose

**FIGURE 10:**
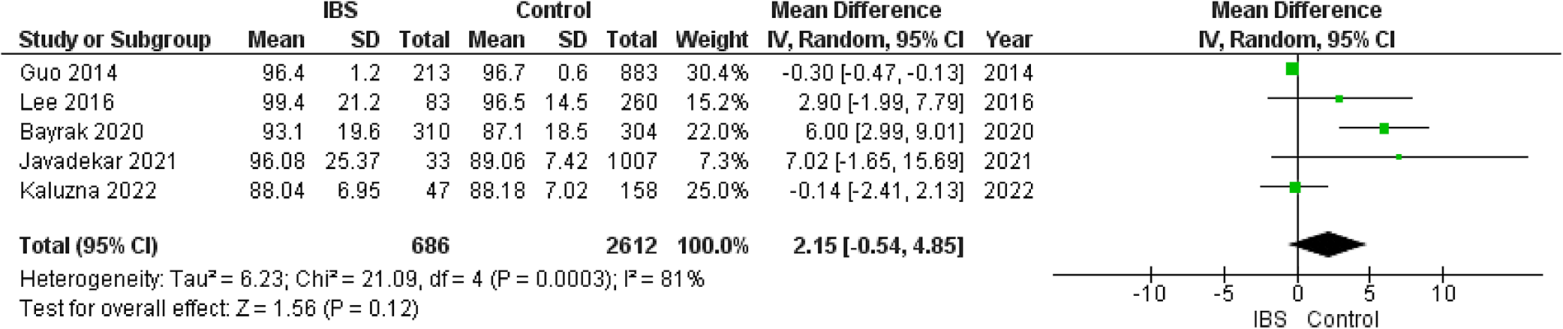
Forest plot of the comparison between the IBS group and control group in fasting blood glucose.

The pooled analysis showed no statistically significant difference between the IBS group and the control group regarding fasting blood glucose (MD = 2.15, 95% CI = −0.54–4.85, *p*‐value = 0.12), with significant heterogeneity among the studies (*p* = 0.0003, *I* ^2^ = 81%). It was solved by a leave‐one‐out test by removing Bayrak (2020) (*p* = 0.22, *I* ^2^ = 32%), and the analysis showed no statistically significant difference between the IBS group and the control group regarding fasting blood glucose (MD = 0.14, 95% CI = −1.23 to 1.52, *p*‐value = 0.84). Figure 10.

#### 3.8.5 HOMA‐IR

**FIGURE 11:**
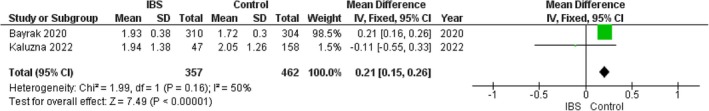
Forest plot of the comparison between the IBS group and control group in HOMA‐IR outcome.

The pooled analysis showed a statistically significant association between the IBS group and increased HOMA‐IR compared with the control group (MD = 0.21, 95% CI = 0.15–0.26, *p*‐value < 0.00001), with no significant heterogeneity among the studies (*p* = 0.16, *I* ^2^ = 50%). Figure 11.

### 3.9 Hypertension

#### 3.9.1 Systolic Hypertension

**FIGURE 12:**
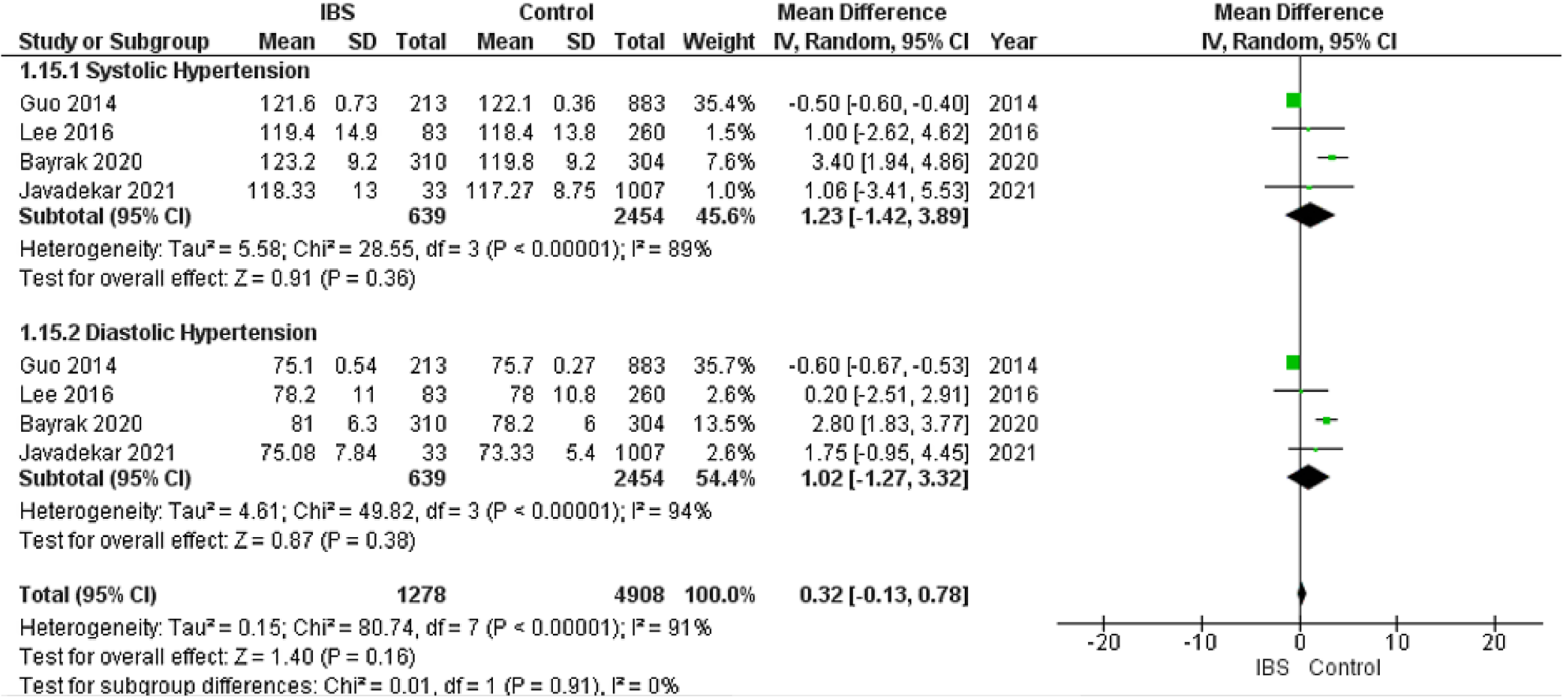
Forest plot of the comparison between the IBS group and control group in the hypertension subgroups.

The pooled analysis showed no statistically significant difference between the IBS group and the control group regarding systolic hypertension (MD = 1.23, 95% CI = −1.42 to 3.89, *p*‐value = 0.36) with significant heterogeneity among the studies (*p* < 0.00001, *I* ^2^ = 89%). It was solved by the leave‐one‐out test by removing Guo (2014) (*p* = 0.34, *I* ^2^ = 8%), and the analysis showed a statistically significant association between the IBS group and increased systolic hypertension compared with the control group (MD = 2.79, 95% CI = 1.32–4.25, *p*‐value = 0.0002). Figure 12.

#### 3.9.2 Diastolic Hypertension

The pooled analysis showed no statistically significant difference between the IBS group and the control group regarding diastolic hypertension (MD = 1.02, 95% CI = −1.27 to 3.32, *p*‐value = 0.38) with significant heterogeneity among the studies (*p* < 0.00001, *I* ^2^ = 94%). It was solved by the leave‐one‐out test by removing Bayrak (2020) (*p* = 0.20, *I* ^2^ = 38%), and the analysis showed no statistically significant difference between the IBS group and the control group regarding diastolic hypertension (MD = −0.09, 95% CI = −1.33 to 1.15, *p*‐value = 0.89). Figure 12.

### 3.10 Lipid Profile

#### 3.10.1 High Density Lipoprotein Cholesterol (HDL‐ C)

**FIGURE 13:**
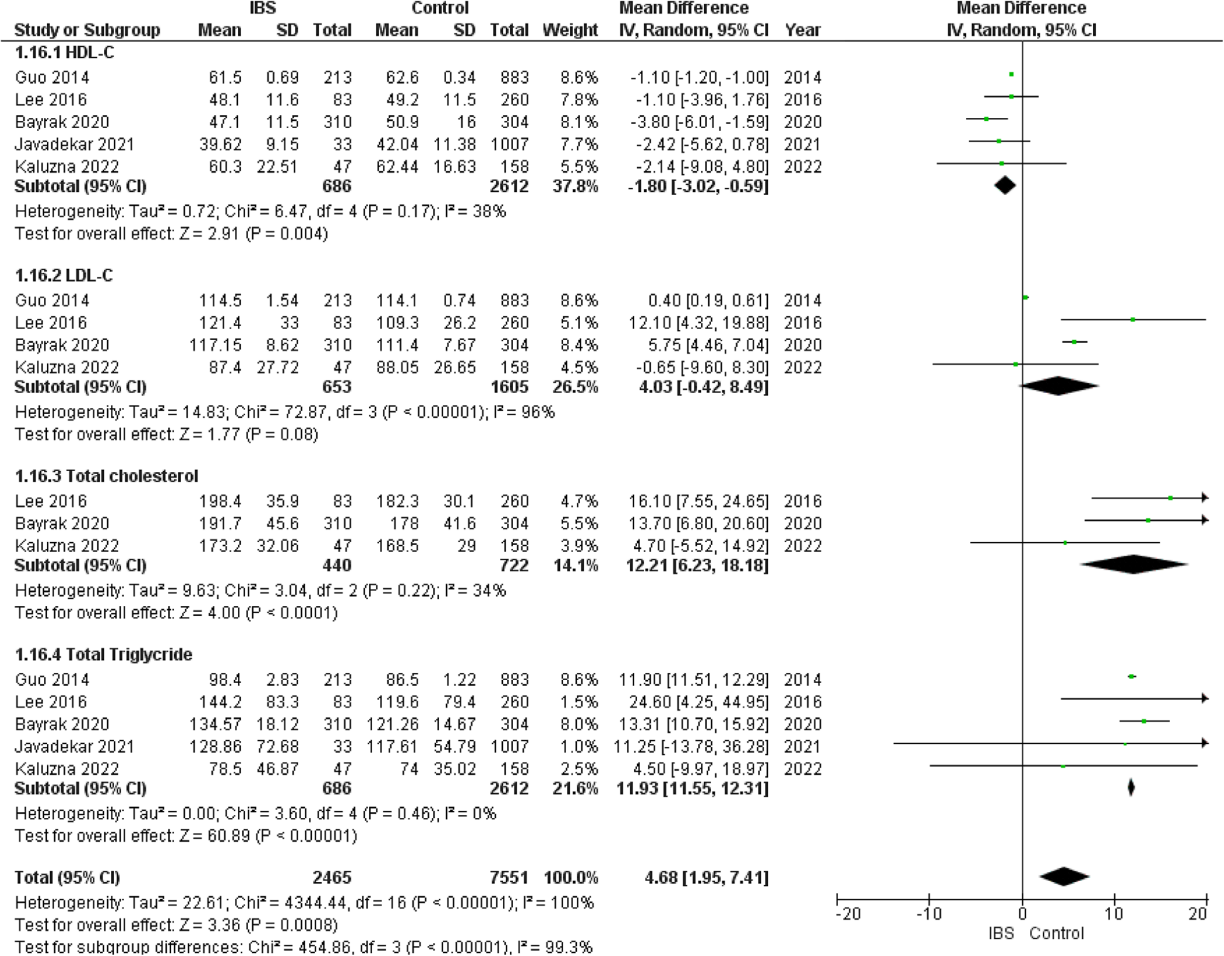
Forest plot of the comparison between the IBS group and control group in lipid profile.

The pooled analysis showed a statistically significant association between the IBS group and decreased HDL‐C compared with the control group (MD = −1.80, 95% CI = −3.02 to −0.59, *p*‐value =0.004) with no significant heterogeneity among the studies (*p* = 0.17, *I* ^2^ = 38%). Figure 13.

#### 3.10.2 Low Density Lipoprotein Cholesterol (LDL‐ C)

The pooled analysis showed no statistically significant difference between the IBS group and the control group regarding LDL‐C (MD = 4.03, 95% CI = −0.42 to 8.49, *p*‐value = 0.08), with significant heterogeneity among the studies (*p* < 0.00001, *I* ^2^ = 96%). It was solved by the leave‐one‐out test by removing Guo (2014) (*p* = 0.10, *I* ^2^ = 56%), and the analysis showed a statistically significant association between the IBS group and increased LDL‐C compared with the control group (MD = 5.98, 95% CI = 0.91–11.05, *p*‐value = 0.02). Figure 13.

#### 3.10.3 Total Cholesterol (TC)

The pooled analysis showed a statistically significant association between the IBS group and the increased total cholesterol compared with the control group (MD = 12.21, 95% CI = 6.23–18.18, *p*‐value < 0.0001), with no significant heterogeneity among the studies (*p* = 0.22, *I* ^2^ = 34%). Figure 13.

### 3.11 Total Triglyceride (TG)

The pooled analysis showed a statistically significant association between the IBS group and the increased total triglyceride compared with the control group (MD = 11.93, 95% CI = 11.55–12.31, *p*‐value < 0.00001), with no significant heterogeneity among the studies (*p* = 0.46, *I* ^2^ = 0%). Figure 13.

## 4 Discussion

Our results depict a significant association between IBS and increased risk of metabolic syndrome. Increased incidence of metabolic syndrome was found among all the IBS subgroups, including the IBS‐constipation, IBS‐diarrhoea, IBS‐mixed, and IBS‐undetermined subgroups. Regarding individual components of MS, we detected a significant association between IBS, obesity, waist circumference, and increased BMI. No statistically significant difference was found between IBS, diabetes, and fasting blood glucose. However, we observed an association with increased HOMA‐IR, an indication of increased insulin resistance. In addition, IBS was found to be associated with increased systolic hypertension. Lastly, our analysis of the lipid profile showed increased LDL‐C, total cholesterol, and triglycerides, and decreased HDL‐C among IBS patients.

Concerning the current literature and its accordance with our results, Evangelos et al. [25] reported a statistically significant association between IBS and MS. On the other hand, Ching‐Sheng et al. [26] found no significant association between IBS and MS.

Regarding the risk of increased body weight among IBS patients, our analysis showed increased body weight in the IBS group compared with the control group. This comes in line with the findings of Monteiro de Mendoca et al. [27] and Evangelos et al. [25] who concluded that IBS patients had increased waist circumference and BMI. Similarly, Aro et al. [28] and Svedberg et al. [29] have concluded that IBS patients had a higher risk of obesity. On the contrary, Tessa et al. [30] and Locke et al. [31] suggested no significant association between increased BMI, obesity, and IBS.

Our analysis of the lipid profile demonstrated a significant increase in TC, LDL‐C, and TG levels, while HDL‐C levels were significantly decreased among IBS patients. This supports the findings of Roczniak et al. [32] who concluded that IBS patients had lower levels of HDL‐C and higher levels of TG; however, their study did not find a significant association between TC, LDL‐C, and IBS. Another study conducted by Gulcan et al. [33] stated that high LDL‐C is seen among IBS patients compared to non‐IBS patients, supporting our findings. Nevertheless, their analysis of HDL‐C and TC showed increased levels among the IBS group and found an insignificant association between TG levels and IBS. This study was limited by the small sample size, which we overcame by analysing the findings of 49,662 individuals.

Although our analysis demonstrated increased insulin resistance among IBS patients, it was not severe enough to cause a significant increase in the fasting blood glucose levels or an increase in the prevalence of diabetes in the IBS group. This reinforces the findings of Kurotani et al. [34] who found an insignificant difference between fasting blood glucose levels in IBS patients compared with the controls, regardless of gender or age groups.

IBS patients had a higher prevalence of systolic hypertension according to our results. This comes in line with the findings of Levine et al. [35] and Dmitry et al. [36] who observed higher values of systolic blood pressure among IBS. On the other hand, Van der Veek et al. [37], Kristy et al. [38], and Gupta et al. [39] concluded an insignificant association between blood pressure and IBS. The increased prevalence of systolic hypertension among IBS patients can be attributed to the role of stress in the pathophysiology of diseases. Psychological stress leads to the alteration of the brain‐gut axis, dysbiosis, and visceral hypersensitivity [40, 41, 42, 43]. A recent study has shown that participants who reported stressful life events had higher systolic blood pressure compared to those who did not [44]. Another study investigated the potential benefit of irbesartan administration, an angiotensin receptor blocker (ARB), among mice where irbesartan led to a decline in intestinal inflammation [45]. Further clinical trials are needed to investigate how ARBs could help treat IBS, especially with concomitant risk of developing hypertension later on. It is important to note that in our analysis of diastolic blood pressure, no significant association was detected between IBS and diastolic hypertension; supporting the findings of Levine et al. [35] and Kristy et al. [38], while Dmitry et al. [36] observed a significant rise in diastolic blood pressure among IBS. Our findings could be clarified by observing the results of a cross‐sectional study that demonstrated the absence of arterial stiffness measured by carotid‐intima thickness among IBS patients and that IBS does not lead to the acceleration of atherosclerosis [46].

### 4.1 Strengths

To our knowledge, this is the first meta‐analysis conducted on the association between IBS and MS. Moreover, our study included a sample size of 49,622 individuals spanning eight countries, enabling us to increase the generalisability of our findings. Lastly, we analysed all components of metabolic syndromes separately to be able to draw more specific conclusions.

### 4.2 Limitation

The majority of included studies was cross‐sectional studies that did not allow us to establish causality. To address this, further prospective studies with long follow‐up durations are needed, investigating the risk of developing MS among IBS patients and whether IBS should be considered an independent risk factor for MS.

## 5 Concluding Remarks

The current investigation suggests an increase in the occurrence of metabolic syndrome among individuals with irritable bowel syndrome (IBS) and its subgroups, in comparison to the control participants. Additionally, when examining the association between IBS and the constituents of metabolic syndrome—namely BMI, obesity, waist circumference, diabetes, fasting blood glucose, high blood pressure, and components of the lipid profile—varying outcomes were observed. However, the significant heterogeneity observed across the included studies limits the generalisability of these findings and warrants cautious interpretation. While these results highlight the need for further investigation, larger‐scale studies with standardised methodologies and diverse populations are essential to validate these associations. Until then, clinicians may consider the possibility of metabolic syndrome among individuals with IBS and explore appropriate preventive and management strategies on a case‐by‐case basis.

## Author Contributions

Y.E.D.: screening, data extraction, writing, and review. S.S.R.: statistical analysis. J.J.L.P.: screening, data extraction, writing, and review. L.L.N.: screening and data extraction. A.A.: screening and data extraction. A.A.: screening and data extraction. F.A.: screening and data extraction. A.A.G.: screening and data extraction. V.M.P.: screening and data extraction. Y.G.: screening and data extraction. K.R.M.: statistical analysis. M.L.P.L.: screening and data extraction. A.G.: screening. L.C.: screening and data extraction. R.A.: screening and data extraction. A.S.: writing and review. L.A.: writing and review. A.S.: writing and review. N.A.: writing and review. A.N.: writing and review. T.M.: writing and review. Y.H.: supervision and correspondence. H.A.: supervision and writing and review. H.A.: conceptualization, supervision, writing, and review.

## Ethics Statement

The authors have nothing to report.

## Consent

The authors have nothing to report.

## Conflicts of Interest

The authors declare no conflicts of interest.

## Supporting information


Appendix S1.

## Data Availability

The data that support the findings of this study are available from the corresponding author upon reasonable request.
