# Supplementary material for: The Relationship Between Irritable Bowel Syndrome and Metabolic Syndrome: A Systematic Review and Meta‐Analysis of 49,662 Individuals
Source: Endocrinol Diabetes Metab. 2025 Mar 24;8(2):e70041. doi: 10.1002/edm2.70041 (PMC11932164; doi:10.1002/edm2.70041)
Supplement: Supplementary file 1 — Appendix S1. [file EDM2-8-e70041-s001.pdf]

PubMed (460)
(IBS[Title/Abstract] OR "irritable bowel syndrome"[Title/Abstract] OR "irritable bowel"[Title/Abstract] OR "Irritable colon"[Title/Abstract] OR "functional bowel"[Title/Abstract] OR "spastic colon"[Title/Abstract]) AND ("Metabolic Syndrome" OR "Abdominal Obesity metabolic syndrome" OR "insulin resistance syndrome" OR "syndrome X" OR "cardiometabolic" OR "Metabolic Cardiovascular" OR "Cardiovascular Syndrome" OR "Dysmetabolic Syndrome" OR "Obesity" OR "overweight" OR "body composition" OR "waist circumference" OR "hip circumference" OR "waist-to-hip ratio" OR "body mass index" OR "diabetes mellitus" OR "fasting plasma glucose" OR "glycosylated hemoglobin" OR "HbA1c" OR "Hypertension" OR "blood pressure" OR "dyslipidemia" OR "lipid profile" OR "low-density lipoprotein" OR "total cholesterol" OR "LDL cholesterol" OR "HDL cholesterol" OR "triglycerides") AND (Healthy OR control OR controlled OR Non-IBS OR "Non-irritable bowel" OR "Non-Functional bowel" OR "Normal bowel")

Scopus (1307)
(IBS OR "irritable bowel syndrome" OR "irritable bowel" OR "Irritable colon" OR "functional bowel" OR "spastic colon") AND ("Metabolic Syndrome" OR "Abdominal Obesity metabolic syndrome" OR "insulin resistance syndrome" OR "syndrome X" OR "cardiometabolic" OR "Metabolic Cardiovascular" OR "Cardiovascular Syndrome" OR "Dysmetabolic Syndrome" OR "Obesity" OR "overweight" OR "body composition" OR "waist circumference" OR "hip circumference" OR "waist-to-hip ratio" OR "body mass index" OR "diabetes mellitus" OR "fasting plasma glucose" OR "glycosylated hemoglobin" OR "HbA1c" OR "Hypertension" OR "blood pressure" OR "dyslipidemia" OR "lipid profile" OR "low-density lipoprotein" OR "total cholesterol" OR "LDL cholesterol" OR "HDL cholesterol" OR "triglycerides") AND (Healthy OR control OR controlled OR Non-IBS OR "Non-irritable bowel" OR "Non-Functional bowel" OR "Normal bowel")

WOS (1027)
(IBS OR "irritable bowel syndrome" OR "irritable bowel" OR "Irritable colon" OR "functional bowel" OR "spastic colon") AND ("Metabolic Syndrome" OR "Abdominal Obesity metabolic syndrome" OR "insulin resistance syndrome" OR "syndrome X" OR "cardiometabolic" OR "Metabolic Cardiovascular" OR "Cardiovascular Syndrome" OR "Dysmetabolic Syndrome" OR "Obesity" OR "overweight" OR "body composition" OR "waist circumference" OR "hip circumference" OR "waist-to-hip ratio" OR "body mass index" OR "diabetes mellitus" OR "fasting plasma glucose" OR "glycosylated hemoglobin" OR "HbA1c" OR "Hypertension" OR "blood pressure" OR "dyslipidemia" OR "lipid profile" OR "low-density lipoprotein" OR "total cholesterol" OR "LDL cholesterol" OR "HDL cholesterol" OR "triglycerides") AND (Healthy OR control OR controlled OR Non-IBS OR "Non-irritable bowel" OR "Non-Functional bowel" OR "Normal bowel")
